# Formation of Thin NiGe Films by Magnetron Sputtering and Flash Lamp Annealing

**DOI:** 10.3390/nano10040648

**Published:** 2020-03-31

**Authors:** Viktor Begeza, Erik Mehner, Hartmut Stöcker, Yufang Xie, Alejandro García, Rene Hübner, Denise Erb, Shengqiang Zhou, Lars Rebohle

**Affiliations:** 1 Helmholtz-Zentrum Dresden-Rossendorf, Institute of Ion Beam Physics and Materials Research, Bautzner Landstrasse 400, 01328 Dresden, Germany; y.xie@hzdr.de (Y.X.); algarc18@ucm.es (A.G.); r.huebner@hzdr.de (R.H.); d.erb@hzdr.de (D.E.); s.zhou@hzdr.de (S.Z.); 2 Faculty of Physics, TU Dresden, 01062 Dresden, Germany; 3 Institute of Experimental Physics, TU Bergakademie Freiberg, Leipziger Str. 23, 09599 Freiberg, Germany; erik.mehner@physik.tu-freiberg.de (E.M.); hartmut.stoecker@physik.tu-freiberg.de (H.S.)

**Keywords:** germanium, germanides, nickel, thin films, sputtering, flash lamp annealing

## Abstract

The nickel monogermanide (NiGe) phase is known for its electrical properties such as low ohmic and low contact resistance in group-IV-based electronics. In this work, thin films of nickel germanides (Ni–Ge) were formed by magnetron sputtering followed by flash lamp annealing (FLA). The formation of NiGe was investigated on three types of substrates: on amorphous (a-Ge) as well as polycrystalline Ge (poly-Ge) and on monocrystalline (100)-Ge (c-Ge) wafers. Substrate and NiGe structure characterization was performed by Raman, TEM, and XRD analyses. Hall Effect and four-point-probe measurements were used to characterize the films electrically. NiGe layers were successfully formed on different Ge substrates using 3-ms FLA. Electrical as well as XRD and TEM measurements are revealing the formation of Ni-rich hexagonal and cubic phases at lower temperatures accompanied by the formation of the low-resistivity orthorhombic NiGe phase. At higher annealing temperatures, Ni-rich phases are transforming into NiGe, as long as the supply of Ge is ensured. NiGe layer formation on a-Ge is accompanied by metal-induced crystallization and its elevated electrical resistivity compared with that of poly-Ge and c-Ge substrates. Specific resistivities for 30 nm Ni on Ge were determined to be 13.5 μΩ·cm for poly-Ge, 14.6 μΩ·cm for c-Ge, and 20.1 μΩ·cm for a-Ge.

## 1. Introduction

The increase of the charge carrier mobility in the channel area of a metal oxide semiconductor field-effect transistor (MOSFET) device is one of the approaches to optimize its performance, especially in terms of the drive current *I_on_* [1,2,3]. Compared with silicon, the electron and hole mobilities in germanium are exceeding those of the commonly used material by a factor of about 2.5 and 4, respectively. Ge was already successfully used in strained Si_1-x_Ge_x_ layers in heterojunction bipolar transistors (HBT) and n-MOSFET-structures [4]. However, the inability to grow a stable high-k Ge oxide layer on Ge together with handling issues of bulk Ge and higher commodity prices have made Ge unattractive for industrial applications for a long time.

In the last two decades, advances in high-k material research and application introduced attractive alternatives to SiO_2_ passivation layers—such as HfO_2_ [5]—and increased the compatibility of Ge for Si-based complementary metal-oxide-semiconductor (CMOS) technology. Thin Ge layers on insulating substrates (GeOI) can be produced by various deposition methods. Two hundred millimeter GeOI wafers, for example, can be manufactured by the Smart Cut technique [6]. Deposition techniques, such as chemical vapor deposition (CVD), magnetron sputtering, or atomic layer deposition (ALD) are widely used as a cheap alternative. For amorphized films, a large variety of thermal treatments can be used to recrystallize them into polycrystalline Ge. One way to classify these methods is to divide them regarding the annealing times. Furnace or ramp annealing is operating in the time range of minutes or hours, while faster methods, e.g., rapid thermal annealing, are in the second to minute range. The third class of annealing methods is ranged in the sub-second time scale. In the case of laser annealing, the time scale is in the order of nanoseconds, whereas flash lamp annealing (FLA) is in the range of 0.1–100 ms [7].

For further integration of Ge into the CMOS process, the formation of ohmic contact materials with sufficiently low resistivity plays a crucial role. Gaudet et al. [8] have identified the most promising candidates for metal–germanide contact materials out of a repertory of 20 metals, by sputter depositing the metals on amorphous Ge (a-Ge) and monocrystalline (100) Ge (c-Ge), respectively. The metal–germanium compounds were formed by ramp annealing. NiGe together with PtGe were found to have the lowest specific resistivity of 22 μΩ·cm. Other results in the range between 13.5 and 17.0 μΩ·cm were reported in literature [9,10,11]. These values are comparable with those of NiSi applied in the CMOS process (17 μΩ·cm [12]). 

Another subject of extensive investigations is the specific contact resistivity between Ge and NiGe, more specifically, the reduction of ohmic losses during charge transport by minimization of contact resistivity, in terms of higher efficiency. The lowest values reported for NiGe on p-type Ge substrates are in the order of 10^−8^–10^−9^ Ω·cm² [13,14], whereas for NiGe on n-type Ge, the values are usually in the range of 10^−7^–10^−8^ Ω·cm² [14,15]. Such discrepancy is due to still prevailing challenges to achieve high active n-type doping levels and the reduction of the Schottky barrier height.

Patterson and Gaudet reported about the formation of a high-ohmic-resistance Ni_5_Ge_3_ phase, appearing in the early annealing stages [8,16]. In other works, Ni-rich Ni_2_Ge and Ni_3_Ge_2_ phases were observed, by applying different annealing methods [17,18]. However, these phases are considered to transform into the orthorhombic NiGe phase in equilibrium processes at elevated temperatures. In contrast, the formation process of Ni–Ge phases under non-equilibrium annealing conditions—in particular under FLA—has not been thoroughly investigated yet. One of the first efforts was made by Prucnal et al. who achieved NiGe formation by using 20 ms flash pulses on 400 nm epitaxially grown Ge films on (100)-Si substrates. The samples with a 50 nm Ni top layer were thermally annealed from the rear side, heating the whole substrate [19]. 

The focus in this work is on the investigation of the NiGe phase formation process under FLA. We will show that with increasing energy density of the flash, the formation starts with the concurrent growth of NiGe and Ni-rich Ni–Ge phases, followed by the transformation of the Ni-rich phases into NiGe. The transformation process is eventually stopped by the depletion of the Ge layer. In addition, we investigate the dependence of the NiGe formation process on the crystallinity of the Ge substrate (amorphous, polycrystalline, or monocrystalline) and on the Ni film thickness.

## 2. Materials and Methods 

Amorphous Ge was deposited from a 3-inch p-type target (99.999% purity, metallic contaminations <10 ppm) by DC magnetron sputtering, in a commercially available sputtering chamber (ROVAK GmbH, Grumbach, Germany), at 60 W sputtering power, on a thermally grown layer of 130 nm SiO_2_ on a 200 μm p-type (100) Si wafer piece with a sample area of 1 cm². The pressure before and during the sputtering process was kept at 5 × 10^−6^ and 5 × 10^−4^ mbar, respectively. The argon flow was set to 10 sccm. To prevent the substrates from unintentional heating during the deposition process and to increase the homogeneity of the film, the sample carrier was commuted with a velocity of 10 mms^−1^ beneath the sputter source. The as-deposited a-Ge thickness was 160 nm. The layer thickness was controlled by ellipsometry measurements in the spectral range of 250–1100 nm. 

To realize thin poly-Ge layers, as-deposited samples were thermally annealed by FLA, using a 100 mm non-commercial tool with a lamp field of 12 xenon-filled flash lamps with an arc length of 250 mm. In order to increase the homogeneity of the spatial light intensity distribution and to maximize the light output, a parabolic mirror is mounted above the lamp field. To achieve different annealing temperatures, the energy density of the flash pulses was varied between 57–64 J·cm^−2^ by varying the applied voltages. The full width at half maximum (FWHM) of the flash pulse was set to 3 ms. According to Raman and atomic force microscopy (AFM) measurements (see Appendix A), the Ge layers are fully crystallized and feature periodic surface structures with a height between 16–23 nm. The resistivity and effective charge carrier concentration of the p-type layers, as derived from sheet resistance measurements, are in the order of 5 and 10^19^ cm^−3^, respectively. 

The Ni layer was sputtered from a 3-inch target (99.95% purity), at 50 W, under comparable conditions on (a) thin a-Ge layers, (b) thin poly-Ge layers, and (c) Sb doped n-type (100) c-Ge wafers with a thickness of 520 µm and a specific resistivity in the order of 1 Ω·cm. Before deposition, the Ge wafers underwent a cleansing process in a bath of a diluted (10 vol. %) hydrofluoric acid (HF) solution. For each of the Ge- substrates 10 nm, 30 nm, and 100 nm of Ni were sputtered, to ensure the reaction of Ni and Ge at different atomic ratios. The thicknesses were controlled by X-ray reflectometry (XRR) measurements.

Immediately after deposition, the layer systems were subsequently annealed by FLA with energy densities between 19–66 J·cm^−2^ in a N_2_ flooded chamber at ambient pressure. This approach allows to study the growth and change of different Ni–Ge phases with respect to their electrical and structural properties during the various annealing steps. For the estimation of the temperature evolution and the peak temperature (*T_peak_*) of the film surface in the time range of milliseconds, simulations were performed using the COMSOL Multiphysics™ software. Details about such kind of temperature simulations are described in [20,21]. 

Raman spectra using a laser wavelength of 532.14 nm, measured with a Jobin Yvon LabRAM HR 800 spectrometer (Kyoto, Japan), was recorded in order to monitor the crystallization for different annealing temperatures. Additionally, AFM was performed on a Bruker Multimode 8 (Bruker Corp., Billerica, MA, USA) tool, to investigate the surface morphology of the Ge-layers. For the structural analysis of the Ni–Ge films, X-ray diffraction patterns were recorded by a Bruker D8 Advance Diffractometer (Billerica, MA, USA), in asymmetric parallel beam geometry at a shallow glancing angle of 0.7° (Grazing Incidence Diffraction) using the Cu Kα wavelength *λ* = 1.5418 Å. Rietveld analysis was performed to identify the origin of the Bragg peaks. To obtain the morphology and element distributions after annealing, high-angle annular dark-field scanning transmission electron microscopy (HAADF-STEM) imaging and spectrum imaging analysis based on energy-dispersive X-ray spectroscopy (EDXS) were performed in a Talos F200X transmission electron microscope (FEI, Brno, Czech Republic) operated at an accelerating voltage of 200 kV. Random orientation Rutherford backscattering spectroscopy (RBS) measurements with He^+^, E = 1700 keV and an incidence angle of 0° were carried out to monitor the element composition in the deposited films. The film sheet resistance was measured by the four-point-probe method, on a semi-automated CMT-SR2000N sheet resistance measurement system (MDC Corp. S.A., Founex, Switzerland), in linear contact alignment and averaged over 30 measurements. The free charge carrier mobilities and concentrations were obtained by using Hall measurements with Van-Der-Pauw geometry. Therefore, a Lakeshore Hall measurement system, with a 775 HMS Matrix was used (Westerville, OH, USA). All electrical characterizations were performed at room temperature conditions.

## 3. Results and Discussion

### 3.1. Electrical Characterization

**Figure 1:**
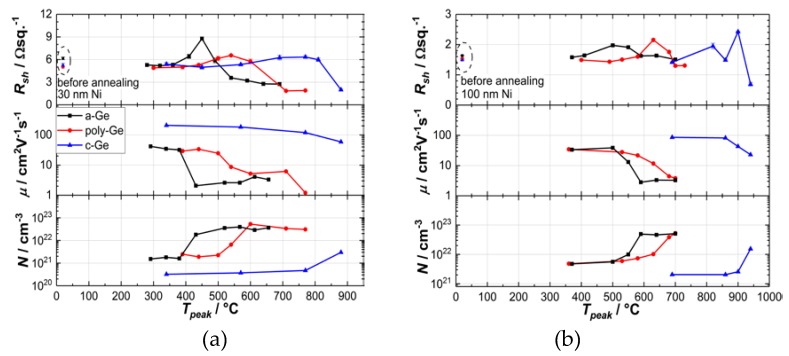
Electrical characterization of annealed Ni/Ge structures for (**a**) 30 nm Ni and (**b**) 100 nm Ni as a function of *T_peak_*. Sheet resistance, mobility and charge carrier density were estimated by four-point-probe and Hall measurements. The carrier concentration was obtained by multiplying the 2D carrier concentration by the effective thickness of the initial Ni/Ge layer system or its equivalent in case of c-Ge.

Sheet resistances *R*_sh_, charge carrier mobilities *µ* and carrier densities *N* are given for the 30 nm and 100 nm thick Ni layers as a function of the annealing temperature in Figure 1a,b), respectively. According to Hall- and four-point-probe measurements, a similar evolution of *R_sh_* was observed for both Ni layer thicknesses during the annealing procedure. The measured data for all Ge substrates, shown in the graphs, can be divided into three characteristic stages on the temperature scale. The first stage is showing a plateau with almost no change in *R_sh_* compared to samples before annealing (data points at 20 °C). The second region features a peak, where *R_sh_* is reaching its maximum values. The decline to values, which are significantly lower than those without annealing, was assigned to the third stage. 

However, a temperature shift between the *R_sh_* profiles can be observed in Figure 1a as well as in Figure 1b, whereby for the reaction of Ni with the a-Ge layer lower temperatures are required than for poly-Ge and c-Ge suggesting different diffusion parameters and -paths for Ni in distinct Ge substrates.

The overview of samples with the lowest measured *R_sh_* for each Ni thickness is displayed in Table 1. The specific resistivity of the NiGe layers can be calculated by multiplying *R_sh_* by the actual stoichiometric NiGe layer thickness, if known. The carrier mobility is decreasing below 10 cm²(Vs)^−1^ for a-Ge and poly-Ge, as *R_sh_* and the carrier density are increasing. It should be mentioned that the carrier concentration was calculated assuming the nominal initial Ni/Ge layer thickness before annealing. The evaluation of the NiGe layer thickness will be discussed in Section 3.2 in more detail.

In the first stage, the contribution of the unreacted Ni layer to the total resistance is assumed to dominate by taking the parallel resistance model into account [22]. Furthermore, the specific resistivity of the Ni layers is two and six orders of magnitude lower than that of the poly-Ge and a-Ge layer, respectively. This fact was verified by measuring the resistivities of 10, 30, and 100 nm Ni films sputter deposited on SiO_2_/Si substrates. The presence of remaining unreacted Ni was monitored by considering the additional contribution of the anomalous Hall Effect (AHE), caused by the ferromagnetic nature of Ni to the Hall-resistance. The determination of *µ* and *N* requires the separation of the ordinary Hall coefficient from the anomalous Hall coefficient by evaluation of the measured Hall resistance in saturation [23]. These saturation points were not reached for the Ni on c-Ge samples during the measurement and thus, the mobility and concentration values are exhibiting rather high uncertainties. Although the unreacted Ni film is still present in the second stage according to the AHE measurements, the gradual consumption of the Ni film is strongly indicated by increasing *R_sh_* values, implying either the formation of a high-resistance Ni-rich germanide phase or a closed layer was not formed by the Ni–Ge phases. In the third stage, no AHE was measured, suggesting the full consumption of Ni. The decrease of *R_sh_* with increasing annealing temperature, in combination with the diminishing unreacted Ni in the third stage, is suggesting a subsequent transformation of the high-resistance, Ni-rich phases into the thermodynamically stable, low-resistance stoichiometric NiGe phase. 

**Figure 2:**
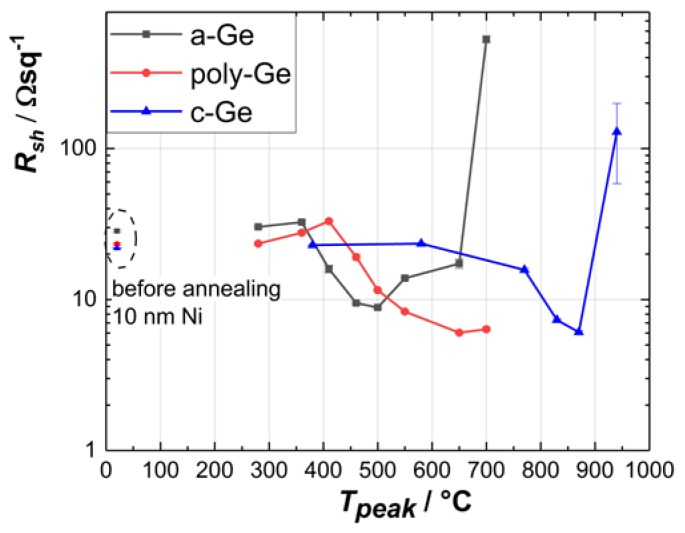
Sheet resistance of flash lamp annealing (FLA)-treated Ni/Ge structures with 10 nm Ni.

The sheet resistance *R*_sh_ of the annealed films with 10 nm Ni (see Figure 2) is showing a steep increase at high temperatures by more than one order of magnitude. This behavior was already described in different works [9,24] and can be explained by the formation of separated NiGe islands at higher annealing temperatures. This explanation is supported by several facts. Firstly, according to the phase diagram for the Ni–Ge binary system, no thermodynamically stable Ni*_x_*Ge_1-*x*_ phase exists in the Ge-rich state (*x* < 0.5) [25,26]. Secondly, the measured *R*_sh_ at high annealing temperature is in accordance with that of poly-Ge films without Ni. A comparable result was also published by Zhang et al., in 2005 [27]. The separation of the NiGe layer and the degradation of its electrical conductivity have made the Hall Effect measurements not expedient for this layer configuration.

### 3.2. Structural Characterization

**Figure 3:**
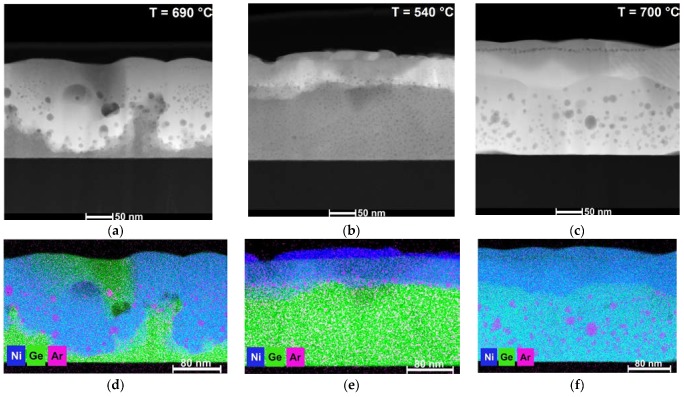
Cross-sectional high-angle annular dark-field scanning transmission electron microscopy (HAADF-STEM) images of annealed bilayers of (**a**) 30 nm Ni on 160 nm a-Ge, (**b**) 30 nm Ni on 160 nm poly-Ge and (**c**) 100 nm Ni on 160 nm poly-Ge at various peak temperatures. The corresponding qualitative element distributions were determined by energy-dispersive X-ray spectroscopy (EDXS) based on spectrum imaging analysis (**d**–**f**).

Cross-sectional HAADF-STEM imaging and corresponding element distribution analysis based on EDXS were performed on selected samples, shown in Figure 3, to exemplify the transformation of the initial layer system. In Figure 3a,d, an annealed sample with 30 nm Ni on a-Ge with *T_peak_* = 690 °C, attributed to the third stage in Figure 1a, exhibits an inhomogeneous distribution of NiGe and poly-Ge. Since the effective layer is showing low-resistance behavior, it is nevertheless to be assumed that the NiGe phase is sufficiently closed. The metal-induced crystallization for the Ni/a-Ge system was investigated by Knaepen et al. [29]. They found that a Ni–Ge phase was formed prior to crystallization with small germanide agglomerates migrating through the a-Ge layer leaving it crystallized behind. Both processes may explain the inhomogeneous distribution of NiGe and poly-Ge phases. Furthermore, a rough, wavy surface is clearly seen in the micrographs. The layer thickness was determined to be 195 ± 10 nm. It was used to calculate the effective layer resistivity, which is varying within 51–55 μΩ·cm. 

The microstructure of 30 nm Ni on 160 nm poly-Ge after FLA at 540 °C, attributed to the second stage in Figure 1a, is shown in Figure 3b,e. Small parts of a remaining unreacted Ni layer were observed during the EDX analysis. Moreover, a 45 nm closed and homogeneous Ni-rich layer was formed with a calculated Ni–Ge ratio of ca. 2:1. The determined element ratios are exhibiting an uncertainty of around 3 at. %. Simultaneously, the growth of small NiGe agglomerations was observed at the Ni-rich Ni–Ge/Ge interface.

Full Ge consumption was observed in Figure 3c,f for 100 nm Ni on poly-Ge after FLA at ca. 700 °C, leaving two Ni–Ge phases behind. The top layer consists of phase with a Ni–Ge ratio of roughly 3:2, followed by the stoichiometric NiGe phase. In this case, the excess supply of Ni prevents the complete transformation of Ni/Ge into stoichiometric NiGe. The measured effective film thickness of 211 ± 5 nm is leading to an upper limit of the resistivity of 27.1–28.3 μΩ·cm.

Considering the full consumption and transformation of Ni into a NiGe layer, one can calculate the theoretical thickness of the NiGe layer and thus the specific resistivities by taking its atomic densities and unit cell volumes into account. In case of 10 and 30 nm Ni, the NiGe layer thickness is 24 and 73 nm, respectively. The resulting resistivities are displayed in Table 1. For the 100 nm samples, the amount of Ni is exceeding that of Ge, which is why the assumption of the full transformation of Ni into NiGe is not valid in this case.

The dark-grey spots seen in all HAADF-STEM images could be identified via EDXS as argon agglomerations. According to RBS data (not shown in this work), the argon contamination of all measured films is in the order of 1 at. %, due to the deposition method. The difference in size and distribution of the bubbles is correlating with the annealing temperature of the layer system. The majority of the argon agglomerations is located in the NiGe layer or at the NiGe/Ge interfaces. 

**Figure 4:**
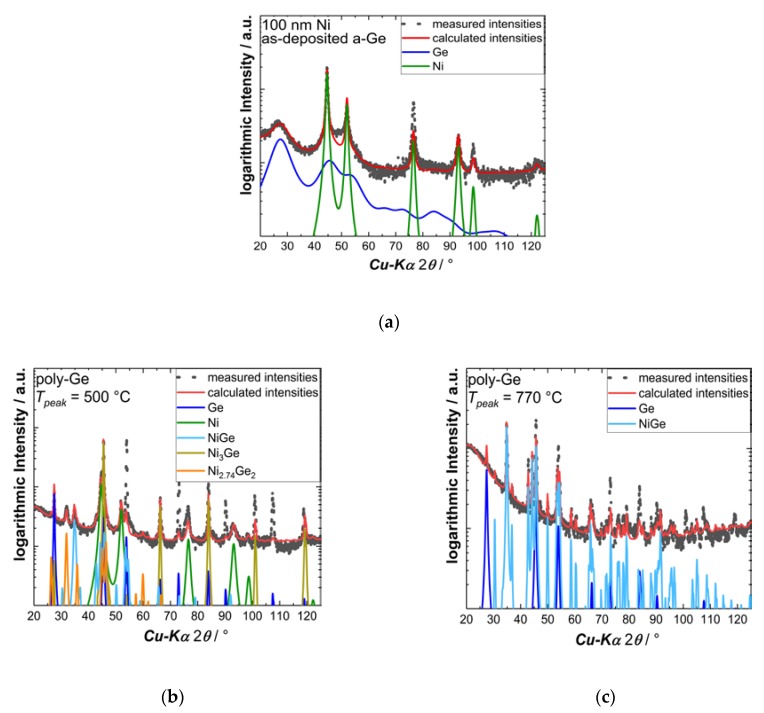
Grazing incidence X-ray diffraction patterns (black points) and Rietveld fits (red) for: (**a**) as-deposited 100 nm Ni on 160 nm a-Ge, (**b**) and (**c**) 30 nm Ni on 160 nm poly-Ge at various peak temperatures. The Rietveld fits are composed of several phases for each sample which are shown in the same color for each sample.

The XRD patterns of annealed 30 nm Ni on poly-Ge and as-deposited a-Ge are exemplarily presented in Figure 4. Patterns of samples in the first stage in are dominated by the Bragg peaks of crystalline Ni, which are accompanied by broad reflections originating from a-Ge and sharp reflections originating from poly-Ge, respectively. The sharp substrate reflections of c-Ge and Si are not observable in the chosen asymmetric geometry. No Ni–Ge phases were observed prior to FLA (see Figure 4a). With increasing annealing temperatures, the Ni signal decreases, indicating the consumption of the Ni layer. However, the temperature at which this happens varies with the type of the Ge substrate, which dictates the onset of the second stage of the annealing process. The Ni signal is disappearing approximately between stage 2 and 3, after *R*_sh_ has reached its maximum. The monoclinic Ni_5_Ge_3_ phase, which was observed in numerous experiments with equilibrium annealing methods, could not be identified in the investigated samples. In fact, hexagonal Ni_2.74_Ge_2_ and cubic Ni_3_Ge are appearing alongside with the orthorhombic NiGe phase between the first and second stage. Further elucidation of this process would require quantification of the found phases through Rietveld-refinement, which is hindered by deviating intensity ratios of the found phases (e.g., in Figure 4b). This, in turn, hints to a growth with preferred orientation of these phases which is not well represented in the used asymmetric diffraction technique. 

After annealing at higher temperature—more specifically beyond stage 2—the Ni-rich phases are receding, whereas the orthorhombic NiGe phase and poly-Ge remain, as shown in Figure 4c. Interestingly, a polycrystalline Ge signal is appearing in case of c-Ge in the region of its melting temperature. This is assumed to be a sign of Ge diffusion from the bulk through NiGe towards the surface. For 100 nm Ni samples, Ni-rich as well as the stoichiometric NiGe phase were observed in both the second and the third stage, due to the fact that all Ge was fully consumed during the annealing process, taking off the possibility for further reactions.

## 4. Conclusions

The phase formation process of NiGe by FLA was investigated. It can be divided into three formation stages. Starting in the first stage with low annealing temperatures, Ni is diffusing into Ge and simultaneously forming Ni-rich and orthorhombic Ni–Ge phases, which are leading to an increase of *R_sh_,* if the fraction of Ni-rich, high-resistance phases is large enough. Cubic Ni_3_Ge and hexagonal Ni_2.74_Ge_2_ were identified among the Ni-rich phases in the case of the 30 nm Ni on poly-Ge substrates. A maximum of *R_sh_* is reached in the second stage, when the Ni layer is separated or completely consumed leaving the Ni-rich layer behind. For even higher annealing temperatures, the Ni-rich phases are transforming into NiGe with a corresponding decrease of *R_sh_* to a final, low-resistance saturation value. According to literature [30], this may happen due to the decomposition of Ni-rich phases into NiGe and Ni, followed by Ni or Ge diffusion into Ge and NiGe, respectively. As poly-Ge was found at the surface in case of c-Ge, Ge diffusion must give a significant contribution in the last stage. In case of a-Ge substrates, the NiGe formation is accompanied by the metal-induced crystallization of Ge leaving an inhomogeneous morphology behind, which has been caused by the migration of NiGe agglomerates into the substrate. 

Finally, NiGe films with low *R_sh_* have been successfully fabricated on poly-Ge or c-Ge by magnetron sputtering and FLA. Assuming that 10 and 30 nm Ni were fully converted into NiGe, the resulting NiGe thickness was calculated to be about 24 and 73 nm, respectively. The NiGe layer thickness estimation was carried out by taking the NiGe unit cell volume and the atomic density of Ni in the Ni layer into account. In the case of 30 nm, the calculation is leading to a resistivity of 13.6 and 14.7 µΩ·cm for poly-Ge or c-Ge, respectively. For 10 nm Ni, both poly-Ge and c-Ge are exhibiting values of 14.8 µΩ·cm. With respect to microstructure and the higher resistivity of 20.1 µΩ·cm for 30 nm Ni and 21.7 µΩ·cm for 10 nm Ni on a-Ge, it seems to be more promising to form NiGe layers on crystalline Ge instead of subsequently depositing Ge and Ni and using only one flash.

## Figures and Tables

**Table 1:** Selected results with lowest sheet resistances with corresponding peak temperatures. The specific resistivities (*ρ*) were calculated by using atomic densities and unit cell volumes from [28].

Ge Phase	Initial Ni Layer Thickness/nm	*R_sh_*/Ω·sq^−1^	Calculated *ρ*/μΩ·cm	*T_peak_*/°C
Amorphous	10	8.86 ± 0.17	21.6 ± 0.4	500
	30	2.74 ± 0.19	20.1 ± 1.4	690
	100	1.51 ± 0.03	---	720
Polycrystalline	10	6.04 ± 0.11	14.8 ± 0.3	650
	30	1.84 ± 0.02	13.5 ± 0.2	710
	100	1.31 ± 0.02	---	700
Single-crystalline	10	6.08 ± 0.12	14.9 ± 0.3	870
	30	1.99 ± 0.09	14.6 ± 0.7	880
	100	0.69 ± 0.06	---	940

**Figure A1:**
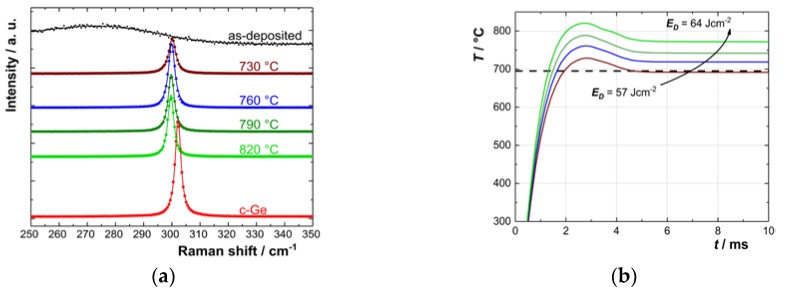
(**a**) Spectra of as-deposited a-Ge, poly-Ge, and c-Ge obtained from Raman scattering measurements. (**b**) Simulation of the annealing temperature of a 1D a-Ge/SiO_2_/Si layer structure for a 3-ms flash pulse. The dashed line indicates the melting point of the a-Ge layer.

**Figure A2:**
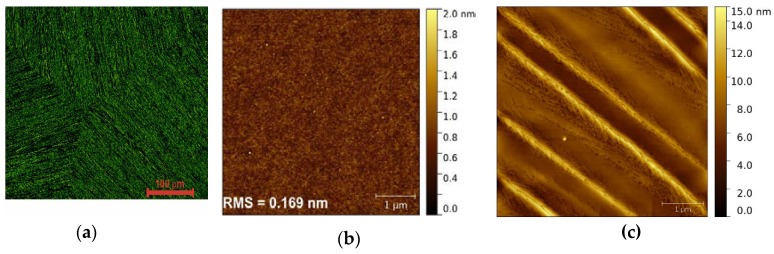
(**a**) Dark-field optical microscopy image of a poly-Ge surface annealed at *T_peak_* = 790 °C. Atomic force microscopy (AFM) scans of (**b**) an as-deposited a-Ge film and (**c**) a poly-Ge film corresponding to the image in (**a**).

## Appendix A

### Polycrystalline Germanium

In Figure A1a, a comparison of a-Ge-, poly-Ge- and c-Ge- Raman scattering spectra is presented. The fitted peak positions and FWHM values are shown in Table A1. The spectrum of a-Ge exhibits a low-intensity broad band with a center at 270 cm^−1^ and no further peaks, especially no peaks from c-Ge. After recrystallization of a-Ge-films via FLA at different applied energy densities, the comparison with the reference c-Ge Raman peak indicates a FWHM broadening accompanied by a peak shift to lower wavenumbers for all poly-Ge samples. The simulated temperature curves at different energy densities are shown in Figure A1b. For all applied energy densities, the simulated peak temperatures (T_peak_) are exceeding the melting point of a-Ge at around 690 °C [31].

It can be assumed, that the recrystallization of a-Ge is taking place from the liquid phase, as suggested by the temperature simulation. Here, it is important to mention that the phase transition into the liquid phase of Ge was not considered in the temperature simulations. According to the measurements, the FWHM of the Ge-related Raman peak is decreasing from 3.0–2.6 cm^−1^, in the temperature range 730–820 °C, while the peaks are slightly shifted to lower wavenumbers at the same time. The origin of the peak shifts is suspected to be caused by tensile strain or smaller grain sizes [32]. In Figure A2a, a dark-field optical microscopy image is presented, exemplarily. The bright periodic structures are separated by grain boundaries. Since the melting point of a-Ge (690 °C) is significantly lower than that of c-Ge (938 °C), the liquid Ge can be considered as an undercooled liquid which is leading to an explosive crystallization (EC) from the liquid phase nucleation (LPN) regime [33]. In the case of the FLA treatment, Ohdaira et al. [34,35] has shown the possibility to crystallize micrometer-thick Ge and Si films through EC.

**Table A1:** Comparison of the Raman scattering spectra of a-Ge and poly-Ge, annealed at different temperatures, and a (100) c-Ge wafer. The peak position and full width at half maximum (FWHM) values were determined by fitting the measured data. The resolution during the measurement was 1.2 cm^−1^.

Phase	Peak Position/cm^−1^	FWHM/cm^−1^	T_peak_/°C
Amorphous	~270	~100	---
Polycrystalline	300.1	3.0	730
	300.1	2.8	760
	299.8	2.6	790
	299.6	2.6	820
Single crystalline	302.2	2.5	---

In Figure A2b, a representative AFM measurement of a sputter deposited a-Ge layer is shown. The RMS of the smooth surface is 0.169 nm. A clear change in the surface morphology is observed after FLA, as shown in panel A2c. The periodic structures, presented as bright lines and corresponding to those found in Figure A2a, were found on every thermally treated sample. They are increasing in height in the range of 16–23 nm as the calculated annealing temperature is increasing from 730–820 °C. No major defects—such as cracks or peeling—could be identified on the processed films. By applying energy densities that lead to temperatures above those covered in this work, full sublimation of the Ge layer is observed. In contrast, energy densities below 57 J·cm^−2^ lead to an incomplete recrystallization with a-Ge islands on the surface.

Sheet resistance measurements were performed directly after deposition and FLA to minimize the influence of natural oxidation of Ge under atmosphere. Specific film resistivity of 160 nm a-Ge was determined to be 69.8 Ω·cm by measuring *R_sh_* and multiplying these values by the known layer thickness. After the annealing step, the resistivity drops several orders of magnitude to 4.8 mΩ·cm at 730 °C and rises slowly up to 6.4 mΩ·cm at 820 °C. The effective charge carrier density was determined to be in the order of 10^19^ cm^−3^. A sharp increase in *μ* (60–100 cm²(Vs)^−1^), together with a decrease in *N*, was observed between 730–760 °C, suggesting a reduction in electrically active lattice defects by i.e. an increase in the grain size [36].
